# Challenges facing the veterinary profession in Ireland: 2. On-farm use of veterinary antimicrobials

**DOI:** 10.1186/s13620-017-0106-9

**Published:** 2017-09-15

**Authors:** Manuel Magalhães-Sant’Ana, Simon J. More, David B. Morton, Alison J. Hanlon

**Affiliations:** 1 Escola Universitária Vasco da Gama, Av. José R. Sousa Fernandes, Campus Universitário - Bloco B, 3020-210 Coimbra, Portugal; 2 School of Veterinary Medicine, University College Dublin, Dublin, Ireland; 3 Centre for Veterinary Epidemiology and Risk Analysis, University College Dublin, Dublin, Ireland; 4 School of BioSciences, University of Birmingham, Birmingham, B15 2TT UK

**Keywords:** Antimicrobial resistance, Focus group, Ireland, One health, Professional ethics, Veterinary ethics, Veterinary prescriptions

## Abstract

**Background:**

Antimicrobial resistance has emerged in recent years as a significant public health threat, which requires both an ethical and a scientific approach. In a recent Policy Delphi study, on-farm use of antimicrobials was a key concern identified by veterinary professionals in Ireland. In this case study (the second in a series of three resulting from a research workshop exploring the challenges facing the veterinary profession in Ireland; the other two case studies investigate clinical veterinary services and emergency/casualty slaughter certification) we aim to provide a value-based reflection on the constraints and possible opportunities for responsible use of veterinary antimicrobials in Ireland.

**Results:**

Using a qualitative focus group approach, this study gathered evidence from relevant stakeholders, namely veterinarians working in public and private organisations, a representative from the veterinary regulatory body, a dairy farmer and a general medical practitioner. Three overarching constraints to prudent on-farm use of veterinary antimicrobials emerged from the thematic analysis: ‘Defective regulations’, ‘Lack of knowledge and values’ regarding farmers and vets and ‘Farm-centred concerns’, including economic and husbandry concerns. Conversely, three main themes which reflect possible opportunities to the barriers were identified: ‘Improved regulations’, ‘Education’ and ‘Herd health management’.

**Conclusions:**

Five main recommendations arose from this study based on the perspectives of the study participants including: a) the potential for regulatory change to facilitate an increase in the number of yearly visits of veterinarians to farms and to implement electronic prescribing and shorter validity of prescriptions; b) a ‘One Health’ education plan; c) improved professional guidance on responsible use of veterinary antimicrobials; d) improved on-farm herd health management practices; and e) the promotion of a ‘One Farm-One Vet’ policy. These findings may assist Veterinary Council of Ireland and other competent authorities when revising recommendations concerning the prudent use of veterinary antimicrobials in farmed animals.

**Electronic supplementary material:**

The online version of this article (10.1186/s13620-017-0106-9) contains supplementary material, which is available to authorized users.

## Background

Antimicrobial Resistance (AMR) has emerged in recent years as a significant threat to public health. The 2016 World Economic Forum Report on Global Risks identifies the rapid and increased spread of infectious diseases (namely due to AMR) as one of the major risks faced by humanity [1]. Numerous reports have highlighted the need for responsible use of antimicrobials [2–4] and using a range of approaches, several countries have substantially reduced national antimicrobial usage in animal production [5–7].

Although the epidemiology of the transfer of AMR between animals and humans is still poorly understood [8], it is known that antimicrobials used to prevent and treat infectious diseases in intensive livestock production systems can contribute to the spread of drug-resistant pathogens in both animals and humans [9]. For example, a connection was found between the use in pigs of third and fourth generation cephalosporins - classified by the World Health Organization as critically important antimicrobials for clinical use in humans [10] - and the increasing prevalence of resistant bacteria [11].

It has been long recognised, at least in developed countries, that the sub-therapeutic use of veterinary antibiotics as growth promoters is morally wrong [12] but only recently has the preventive use of antimicrobials received a similar ethical appraisal [13–15]. Although the use of antimicrobials as growth promoters has been banned in the EU since 2006 (Regulation (EC) No. 1831/2003), current husbandry practices for intensive farming systems often involve preventive antimicrobial use [9]. However, refraining from preventive use of antimicrobials is, in itself, insufficient to fulfil our moral duties regarding antimicrobial stewardship since it should also consider therapeutic decisions. For example, a recent survey showed that European veterinarians prescribe antimicrobials empirically. Antimicrobial sensitivity testing is mostly performed as a result of poor response to initial therapy, with one in 10 veterinarians never performing antimicrobial sensitivity tests before starting antimicrobial treatment [16].

Taken together, these findings are a potential source of reputational damage to the veterinary profession [17] and support the argument that promoting prudent use of antimicrobials, and thus tackling AMR, requires an ethical as much as a scientific approach. Understanding the context and challenges faced by veterinarians in Ireland on antimicrobial usage is required. Within a wider research project on the ethical challenges facing the veterinary profession in Ireland, this is the second of a series of three case studies exploring key issues identified in a recent Policy Delphi consultation process [17]. The other two case studies investigate clinical veterinary services [18] and emergency/casualty slaughter certification [19]. This case study aims to provide a value-based reflection on the constraints and potential opportunities for prudent on-farm usage of veterinary antimicrobials in Ireland.

## Methods

### Sample design

A research workshop to explore the constraints and potential opportunities for prudent on-farm usage of veterinary antimicrobials in Ireland was held at University College Dublin on 18 June 2015. Nine stakeholders agreed to participate, but only eight attended. Purposive sampling of participants was used to reflect the range of roles on the use of veterinary antimicrobials. With one notable exception, the selection process started with the identification by the authors of Irish organisations that were considered stakeholders to the use of veterinary antimicrobials in Ireland (Table 1).

**Table 1:** Organisations that were considered stakeholders to the use of veterinary antimicrobials in Ireland

ORGANISATION	ACRONYM	MAIN ROLE
Department of Agriculture, Food and the Marine	DAFM	Regulator of veterinary medicines, under the European Communities (Animal Remedies) (No. 2) Regulations 2007.
Health Products Regulatory Authority (formerly the Irish Medicines Board).	HPRA	State agency responsible for regulating the safety and quality of medicines, medical devices, and other health products.
Bord Bia (Irish Food Board)	IFB	Body responsible for implementing farm quality assurance schemes in Ireland.
Veterinary Council of Ireland	VCI	Regulator of the veterinary profession.
Animal Health Ireland	AHI	Umbrella agri-food organization (representing veterinarians, farmers, processors, state agencies) responsible for implementing a number of animal health programmes in Ireland.
Food Safety Authority of Ireland	FSAI	Statutory body responsible for food safety and hygiene
Irish Farmers Association	IFA	Organization representing the different sectors of Irish food production.
Veterinary Ireland	VI	Representative body for veterinary surgeons in Ireland

Selection criteria included seniority, experience with the research topic, and an active role with a relevant veterinary organisation. Participants included five veterinarians (working in academia, pharmaceutical industry, Health Products Regulatory Authority, Department of Agriculture, Food and the Marine, and Animal Health Ireland), a representative from the national regulatory body, and a dairy farmer. Participants had mixed experience in clinical practice and in implementing farm quality assurance schemes. A Private Veterinary Practitioner (PVP), and member of Veterinary Ireland, had agreed to participate in the workshop but did not attend. To provide a One Health perspective, a general medical practitioner (GP) actively involved in the prudent use of antimicrobials in human medicine was also invited to participate (Table 2).

**Table 2:** Participants in focus groups regarding on-farm use of veterinary anti-microbials (VAM)

	Gender	Stakeholder
VAM-1	M	Health Products Regulatory Authority
VAM-2	F	Department of Agriculture Food and the Marine
VAM-3	M	General Medical Practitioner
VAM-4	F	Veterinary Council of Ireland
VAM-5	F	Pharmaceutical Industry
VAM-6	M	Animal Health Ireland
VAM-7	F	University College Dublin
VAM-8	M	Irish Farmers Association

### Focus groups

The sessions were moderated by the third author (DBM), who is experienced in chairing working groups, and audio-recorded for qualitative analysis. An interview guide had been developed by the first author (MMS), discussed with co-authors, and revised until final agreement was reached. A semi-structured approach was used to guide the conversation towards the research questions (Additional file 1). The focus group consisted of two consecutive sessions (100 and 125 min duration). In the morning session, each participant was asked to list the three main challenges associated with prescribing and administering antimicrobials, and to share their views with the group as part of a facilitated discussion. This was followed by a vignette, validated elsewhere [20], describing a case scenario involving the prescription of veterinary antimicrobials (Table 3), followed by a discussion on who should take responsibility for the prescription and administration of antimicrobials.

**Table 3:** Vignette, used in focus group session, describing a case scenario on prescription of veterinary antimicrobials

Joan routinely prescribes broad spectrum antibiotics (injectable and tubes) to a dairy farmer with a large herd of 300 animals in Co. Cork. The herd has a low record of somatic cell count (< 100,000 cells/mL). Every dry cow gets a tube and most cows are injected. “The preventive use of antibiotics has made this farm one of the best in Ireland – at the end that’s good for the animals, and cheaper for the farmer.”	

The afternoon session started with the identification of barriers to adequate prescription and dispensing of veterinary antimicrobials and thereafter exploration of possible solutions to improve prudent on-farm antimicrobial use. After the event, a summary with the main conclusions was sent to participants for comment and clarification.

### Data handling and analysis

The sessions were transcribed verbatim, anonymised and inserted into NVivo 10, a qualitative research analysis software (© QSR International 2013). Thematic analysis was conducted using the data immersion/reduction technique proposed by Forman and Damschroder [21]. As an initial deductive step, the research questions were used to sort and categorise the data according to two thematic, predetermined areas (i.e. constraints and opportunities). An inductive approach was then applied and a preliminary list of themes was generated after the initial coding, run by MMS and discussed with the senior author (AJH). The list of themes was refined in the following coding runs, while adding subthemes. The process was repeated iteratively until a final agreement was reached.

## Results

Participants identified a number of stakeholders involved in the use of veterinary antimicrobials in Ireland, namely PVPs, farmers, pharmaceutical companies, pharmacists and retailers/processors, reflecting a shared predicament requiring shared solutions. It was reflected that a collective responsibility may limit individual ownership of the problem because:

*“(…) I accept there is a problem but in a sense it is not my problem. (…) There is a tactic[al] denial that everybody who prescribes or uses antibiotics can contribute to [addressing] the problem but then there is a tendency to say: but my contribution is so minor and so small and so insignificant that actually I don’t need to do anything or change. Because there are other people who have much more influence on the thing than I do and if they change then I will change.”* (VAM-3)

**Fig. 1:**
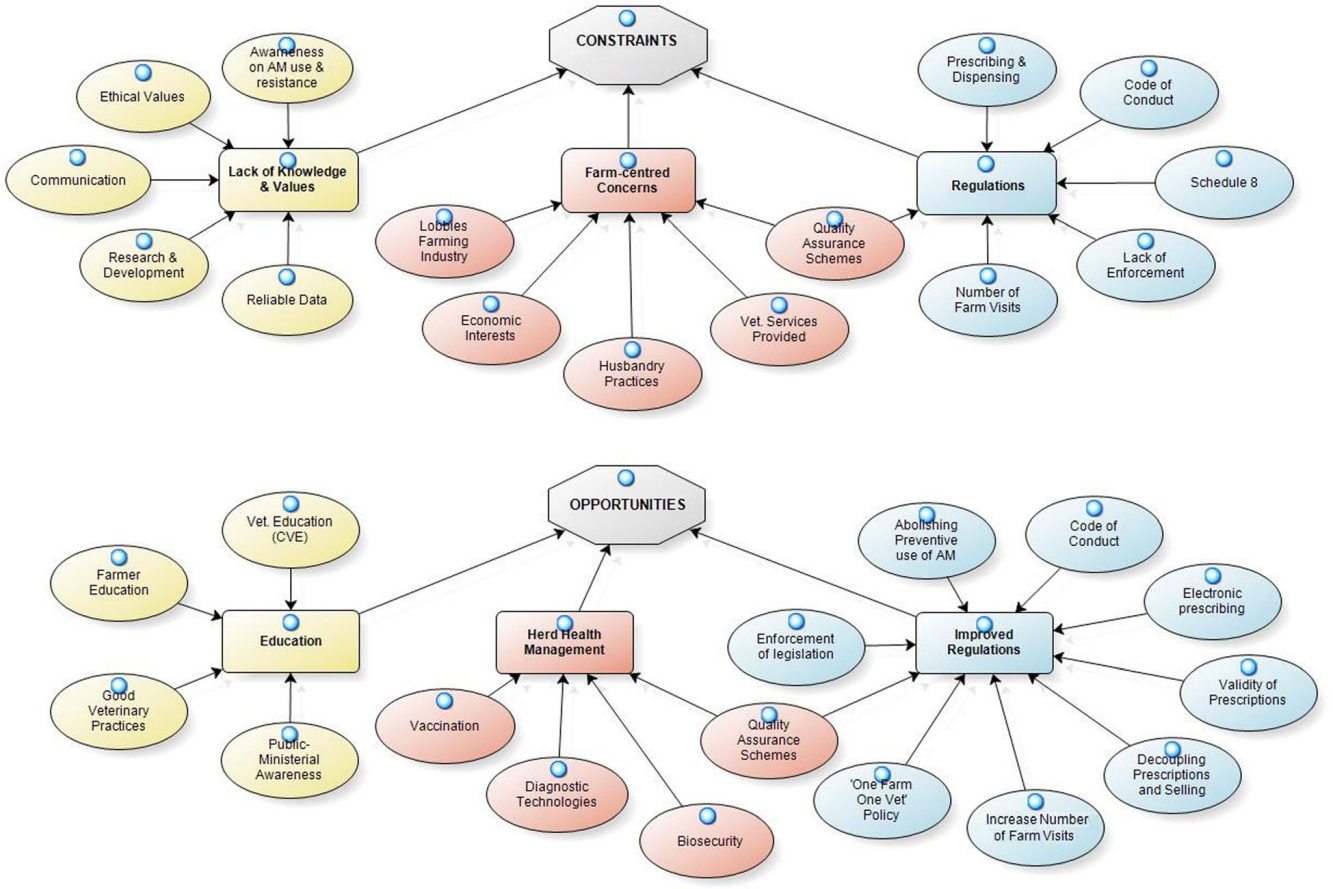
Constraints and opportunities regarding the on-farm use of veterinary antimicrobials in Ireland. Themes and subthemes that emerged from the thematic analysis, including the relationship between them, are represented using NVivo 10 Software

Three overarching constraints to the prudent on-farm use of veterinary antimicrobials emerged from the thematic analysis: ‘Deficiencies in current regulations’, ‘Lack of knowledge and values’ regarding farmers and vets and ‘Farm-centred concerns’, including economic and husbandry concerns. Conversely, three main themes which reflect possible opportunities to the barriers were identified: ‘Improved regulations’, ‘Education’ and ‘Herd health management’ (Fig. 1).

### Improved regulations

In terms of current regulations, it was mentioned that the EC (Animal Remedies) Regulations 2007 does not ensure adequate on-farm clinical surveillance on the basis of ‘at least’ one compulsory veterinary visit a year. In practice, several veterinarians can prescribe and dispense remedies to the same farm resulting in multiple sources of antimicrobials. It was pointed out that a PVP “*can sit in [an Irish county] in his office and write prescriptions all day, every day (…) [which] means that anybody who is a clinician has not got full knowledge of what [remedies] have gone into that unit*” (VAM-5). The suggestion was made to have a “*a minimum of four veterinary visits per year*” (VAM-6) that would promote a change of the role of veterinarians towards “*health consultants as opposed to retailers of medicines*” (VAM-2). Poor enforcement was also acknowledged as a serious constraint since “*it is very hard to get a watertight legislation that will cover every possible scenario”* (VAM-2), together with the difficulty of getting evidence to follow through with legal cases.

Participants were in agreement about the dearth of reliable farm-level data on the use of antimicrobials, and on AMR levels in animals, which impedes the setting of targets for antimicrobial usage. The suggestion was made to endorse electronic prescribing, to facilitate the opportunity for centralised recording, and to reduce their duration of validity, each to promote responsible prescribing. Lack of investment in research and development in antimicrobial testing was also identified as a barrier to enforce legislation, as illustrated by the following example: “*we now know the farm the pig came from* (…)*, but why can’t you link that to how much antimicrobials are there? Trace it right back to the farm and we will penalise it when we find it*” (VAM-8).

It was also suggested that a ‘One Farm, One Vet’ policy should be implemented, giving a single veterinarian full responsibility for a given farm; however, provisions should be made in “*finding the balance where you’ve got responsible use but also you’ve got a livelihood for vets*” (VAM-2). A call for ethical guidance was made, which may promote good veterinary practice and prevent conflicts with the farmer and other colleagues:

“*If I said I'm going to visit [the farm] three times and [the farmer] says I will only pay you once, then someone else is happy to visit once and prescribe three times. (…) I suppose it’s back to the challenge of defining responsible use and legislating for it, be that in State law or in professional code of conduct*.” (VAM-6)

### Education

Increasing the standards of practice would involve improving not only existing guidance on good veterinary practice (e.g. Code of Professional Conduct) but also requirements in terms of Continuing Veterinary Education (CVE). In this regard, the suggestion was made to have a more prescriptive form of CVE where veterinarians would be required to attend courses in their area of speciality or expertise, including the prudent use of antimicrobials. It was argued, however, that the current CVE courses would have to be expanded in order to cover the wide scope of veterinary educational needs.

The connection between the use of antimicrobials in humans and animals was an overarching theme, and participants highlighted the need for a ‘One Health’ educational approach involving all stakeholder groups. In terms of farmer education, it was acknowledged that farmers generally have little awareness of the connection between husbandry practices and the potential for human health issues. In order to effect change, the message that AMR is primarily a human health issue needs to be conveyed to the farmers because “*until they see the link between antimicrobial resistance (..) and their livelihood and their children’s wellbeing (..) you won’t change them*” (VAM-7). Effective messages on public awareness on AMR would need to be conveyed, bearing in mind that “*behavioural change is the hardest thing of all*” (VAM-2).

### Herd health management

It was recognised that improving current husbandry practices is central to decreasing the use of antimicrobials at the farm level. Particular concerns were raised for the pig industry, where antimicrobial usage can be high. Conversely, it was suggested that competing economic interests have hindered the full potential of current farm assurance schemes, particularly with respect to meaningful veterinary oversight. In this respect, it was proposed that retailers and processors could positively promote change by increasing their quality assurance standards, procedures and implementation, thus triggering a ‘virtuous circle’ of improved farming practices.

Other health management measures at farm level were suggested, namely the development of on-farm diagnostic testing, improved biosecurity measures and increased vaccination. The industry participant mentioned how poor adherence to vaccination can be linked to lack of veterinary supervision by saying that *“my message has to go through the vets to encourage more vaccine uptake. And if the vets aren’t there [the farm] in the first place, then my message can’t get to the end user”* (VAM-5).

## Discussion

This study highlights perceived barriers to prudent prescribing and dispensing of veterinary antimicrobials in farm animals in Ireland, and presents possible opportunities for solutions to be developed. By virtue of their responsibilities, veterinarians are professionally and ethically responsible for mitigating the risk of AMR and conserving the therapeutic potential of antimicrobials for future generations [14, 22]. Faced with the value-based decision to administer antimicrobials to farm animals, veterinarians need to consider the range of stakeholders that may be affected, and their often-conflicting interests. This decision-making ability renders veterinarians as independent health consultants as opposed to retailers of medicines. In this regard, antimicrobials have been considered ‘common or public goods’ which need to be managed with fairness and responsible stewardship [13].

In turn this generates an ethical issue, that of an utilitarian responsibility to promote the greatest good for the greatest number and not simply a greater good for a larger number, or even simply a greater good for the few (e.g. immediate stakeholders such as the individual veterinarian and the farmer). It is now obvious that such a ‘good’ is seen as broader than the commercial interest and improved animal health and welfare [23, 24], important as these are, to the problems produced by the increasing ineffectiveness of antibiotics for human health. Consequently, the use of antimicrobials in animals is reminiscent of Garret Hardin’s parable *The Tragedy of the Commons* [25, 26]. In effect, the quotation from participant VAM-3 (cf. results) is well illustrative of this ethical conundrum.

AMR is a global issue, and effective solutions will require a global approach, as illustrated by the *First World Antibiotic Awareness Week* (16–22 November 2015). There are numerous examples highlighting the urgency of this issue, including identification of *Escherichia coli* isolates resistant to colistin (an antimicrobial of last resort in human medicine) in China in 21% of animals tested, 15% of raw meat samples and in 1% of clinical isolates from patients [27]. Substantial work is underway to address this problem. International best practices, namely from The Netherlands [6, 28] and Denmark [5], highlight the critical value of objective measurement of antimicrobial usage, both in terms of on-farm usage and veterinary prescribing, to allow benchmarking, the sanctioning of high users and prescribers, and factual assessment of progress towards agreed national targets. Furthermore, in these countries, additional veterinary controls have been introduced (in Denmark, veterinarians cannot profit from antimicrobial sales; in The Netherlands, farmers are only allowed to engage a single veterinarian) to significantly reduce antimicrobial use in farm animals, while safeguarding productivity. Further, a comprehensive review of strategies to limit the need to use antimicrobials in farm animal production was recently completed by the European Food Safety Authority and the European Medicines Agency [29]. In this report, the authors highlighted the need for a multifaceted and integrated approach, adapted to local conditions. Within the European Parliament, a ban on the preventive use of veterinary antimicrobials in the EU is under consideration [30]. If this were enacted, improved current husbandry practices would be required in the absence of preventive antimicrobial usage, to limit animal health and welfare concerns particularly in intensive livestock production.

In Ireland, some insights into antimicrobial usage are available, from studies with a primary focus on antimicrobial usage [31] and national sales data. The latter have been used in defined studies of intramammary antimicrobial usage [32, 33] and are also reported by the national regulator [34]. Farm-level antimicrobial usage data is not available in Ireland, therefore benchmarking of on-farm usage and veterinary prescribing is not yet possible.

As identified by study participants, one of the main results from the current study is the need for a more robust regulatory system (namely to increase the number of yearly visits of veterinarians to farms, to implement electronic prescribing and shorter validity of prescriptions) together with improved enforcement. These issues were previously highlighted in a report from Veterinary Ireland [35]. Under the European Communities (Animal Remedies) (No. 2) Regulations 2007 (SI No. 786 of 2007, including subsequent amendments), antimicrobial veterinary medicinal products may only be supplied in Ireland on the basis of a prescription from a registered veterinary practitioner “*to a maximum quantity of 12 months supply*” (Regulation 43(b)) and having visited the farm “*at least once in a 12 month period*” (Regulation 43(8b)). This has led to concerns of remote veterinary prescribing [17, 35], which may negatively impact on both prudent on-farm antimicrobial use and the reputation of veterinary profession. Furthermore, in Ireland there is no requirement when prescribing intramammary antimicrobial agents for a farm visit at least once annually, which was a concern raised in the above-mentioned report [35].

The study participants also highlighted a need for improved professional guidance on prudent and responsible use of veterinary antimicrobials within the VCI Code of Professional Conduct (CPC). In effect, the VCI-CPC specifically refers to the use of drugs in competition animals (i.e. animals used in sport) but does not include provisions regarding the prudent and responsible use of antimicrobials [36]. Partially to cover this need, in 2008 the VCI issued the booklet ‘Ethical Veterinary Practice’, providing additional guidance on the responsible prescription and dispensing of remedies to farm animals, and taking the EC (Animal Remedies) Regulations 2007 into account [37]. This booklet (which was due to be incorporated into the VCI-CPC) highlights, but does not resolve, the challenges of prescribing veterinary antimicrobials and would need to be complemented by additional ethical guidance, in the VCI-CPC or elsewhere, namely on the precautionary principle [15, 38] and obligations to future generations [13].

It is known that veterinarians base their prescription decisions on tangible factors, such as patient benefit, client satisfaction and economic drivers, with lesser consideration of wider, and perhaps more abstract notions of AMR and societal good [23, 39]. Moreover, a recent survey showed that Irish veterinary cattle practitioners are more likely to prescribe antimicrobials on ‘owner demand’ (if the farmer wants them or if they think that the farmer expects it), and to avoid being blamed if antimicrobials later prove necessary [40]. Cultural differences in prescribing practices must also be considered. For example, Postma and colleagues found that Dutch veterinarians are less likely to rely on antimicrobials than Flemish veterinarians and seem less pressurised by farmers to do so [41]. However, these results may reflect the stricter policy measures to reduce antimicrobial use that had already been implemented in The Netherlands. Further research will need to be undertaken to understand the drivers influencing antimicrobial prescription in different jurisdictions.

The study participants also recommended the establishment of a national education plan on responsible antimicrobial use in farm animals, encompassing issues relating to both public and animal health (a so-called One Health education plan). For example, an important finding from the discussion group concerned farmers’ perceived lack of knowledge with respect to AMR. This is of broader relevance given the contribution of farm animal antimicrobial usage on AMR, both in animals and people. Further, there is evidence of increased prevalence of AMR bacteria among farmers and others in direct contact with farm animals [42–44].

Although the One Health education plan was only briefly explored during the focus group, the participants recommended that this should target veterinarians, farmers and the public, and probably take into account the drivers and barriers to behaviour change “*to ensure the future universal access, sustainability, and effectiveness of antimicrobials to treat disease in people and their livestock*” ([45], p.3).

The participants emphasised the need for education of veterinarians on good stewardship of antimicrobial drugs [46]. In effect, years in veterinary practice could be related with decreased antimicrobial stewardship; for example Dutch veterinarians were seen to be less concerned with AMR and more inclined to prescribe antimicrobials prophylactically, the more experienced they were [47]. In that regard, CVE on AMR, prudent prescribing and ethical decision-making can contribute to increase awareness and raise standards of practice, especially if veterinarians would be expected to complete a significant portion of their yearly mandatory 20 CVE points within their remit of expertise. Furthermore, van Dijk and colleagues have demonstrated that participatory policy making provides a platform for stakeholders to increase their understanding of the risks associated with routine intramammary antimicrobial usage in dairy cows and enter into voluntary agreements to implement best practice on-farm [48].

There is clear agreement for both global and regional action against AMR. The EU recently released a Community action plan, with key priorities including making the EU a best practice region, boosting research, development and innovation on AMR, and shaping a global agenda [49]. Relevant to farm animal production, EFSA and EMA have recently finalised scientific advice on prudent use of antimicrobials [29], and EMA is progressing work towards harmonised collection of data on consumption of veterinary antimicrobials [50]. Further, each European member states has developed national action plans, including limiting antimicrobial usage in farm animals. A large number of management strategies are relevant, but may vary between farms and regions, including Quality Assurance Schemes [51], biosecurity, vaccination and on-farm diagnostic testing. It is clear that an integrated approach is needed, including improved on-farm management of herd health, ongoing measurement and benchmarking of on-farm antimicrobial usage, and relevant education and training.

Recommendations from this study mirror those of similar working groups [35, 52], but also add new insights. In contrast to these earlier studies, however, participants in the current study remained anonymous and were not expected to reach a consensus, which allowed a wide range of opinions on antimicrobial use to be explored. We note a number of challenges from the suggestions made. For example, the ‘One Farm, One Vet’ policy conflicts with current competition regulations (Competition and Consumer Protection Act 2014). Constructive guidance by the competent authorities such as the VCI should be given that could help circumvent the legislative hurdles that impede veterinary professionals from having full control over the remedies used at the farm level, similarly to that achieved in The Netherlands and following the recommendations from the Federation of Veterinarians of Europe [53]. The ‘One Farm, One Vet’ policy, as is now required in The Netherlands, would ensure that veterinarians in Ireland have a complete understanding of all of the antimicrobials that have and are being used on their clients’ farms. This is not currently possible if there is more than one prescribing veterinarian, each potentially with only a partial understanding of both the animal health and the antimicrobial prescribing situations on these farms.

The present study is part of a wider workshop where participants were divided into three groups, on the grounds of their expertise, and some limitations should be acknowledged. This investigation relied on two focus group sessions and on the same group for both sessions. Whilst five of the eight participants in the focus groups were veterinary professionals, the PVP who had agreed to participate did not attend, and thus limiting the results due to the lack of a prescribing veterinarian. Nonetheless, two participant veterinarians had substantial clinical experience in large animal practice and pig practice, and the group was sufficiently diverse in order to minimise cohort effect. Further, it was the role of the moderator to ensure that every participant had a chance to meaningfully contribute to the debate. Results from this study should be extrapolated with caution since the small number of participants involved in this study is unlikely to represent the full range of views of every stakeholder involved with the use of veterinary antimicrobials in Ireland.

## Conclusions

Five main conclusions can be drawn from this study based on the perspectives of study participants:

- Regulatory change should be promoted in order to increase the number of yearly visits of veterinarians to farms, implement electronic prescribing and shorter validity of prescriptions.
- Better on-farm herd health management practices should be endorsed, such as increased vaccination, the development of on-farm diagnostic testing, improved biosecurity measures and Quality Assurance Schemes.
- A ‘One Health’ education plan should be sought, including targeted veterinary CVE, farmer education and public awareness.
- Guidance on prudent and responsible use of veterinary antimicrobials should be included within the VCI-CPC.
- A forum to discuss the feasibility of a ‘One Farm, One Vet’ policy should be promoted.

It is hoped that these findings will assist the Veterinary Council of Ireland and other competent authorities when revising recommendations regarding the prudent use of veterinary antimicrobials in farmed animals. These findings may also be relevant to regulatory authorities in other countries.

## Additional file

Additional file 1: Interview Guide - FOCUS GROUPS VAM. (PDF 11 kb)

## References

[1] WEF. The Global Risks Report, 11^th^ Ed. 2016. Geneva, Switzerland: World Economic Forum; 2016. https://www.weforum.org/reports/the-global-risks-report-2016. Accessed 4 Jan 2017.

[2] European Commission (2016). Special Eurobarometer 445 - Antimicrobial resistance.

[3] FAO (2016). The FAO action plan on antimicrobial resistance 2016–2020.

[4] WHO. Global action plan on antimicrobial resistance. Geneva, Switzerland: World Health Organization; 2015. http://www.who.int/antimicrobial-resistance/publications/global-action-plan/en/ Accessed 16 Jan 2017.

[5] Aarestrup F (2012). Sustainable farming: get pigs off antibiotics. Nature.

[6] Speksnijder DC, Mevius DJ, Bruschke CJM, Wagenaar JA (2015). Reduction of veterinary antimicrobial use in the Netherlands. The Dutch success model. Zoonoses Public Health.

[7] Sjölund M, Postma M, Collineau L, Lösken S, Backhans A, Belloc C (2016). Quantitative and qualitative antimicrobial usage patterns in farrow-to-finish pig herds in Belgium, France, Germany and Sweden. Prev Vet Med.

[8] Woolhouse MEJ, Ward MJ (2013). Sources of antimicrobial resistance. Science.

[9] Boeckel TPV, Brower C, Gilbert M, Grenfell BT, Levin SA, Robinson TP (2015). Global trends in antimicrobial use in food animals. Proc Natl Acad Sci.

[10] WHO. Critically important antimicrobials for human medicine - 3rd Revision, 2011. Geneva, Switzerland: World Health Organization; 2012. http://www.who.int/foodsafety/publications/antimicrobials-third/en/ Accessed 16 Jan 2017.

[11] Agersø Y, Aarestrup FM, Pedersen K, Seyfarth AM, Struve T, Hasman H (2011). Prevalence of extended-spectrum cephalosporinase (ESC)-producing Escherichia Coli in Danish slaughter pigs and retail meat identified by selective enrichment and association with cephalosporin usage. J Antimicrob Chemother.

[12] Rollin B (2001). Ethics, science, and antimicrobial resistance. J Agric Environ Ethics.

[13] Littmann J, Viens AM (2015). The ethical significance of antimicrobial resistance. Public Health Ethics.

[14] Littmann J, Buyx A, Cars O (2015). Antibiotic resistance: an ethical challenge. Int J Antimicrob Agents.

[15] Scott HM, Midgley G, Loneragan GH (2015). Antimicrobials in animal agriculture: parables and policy. Zoonoses Public Health.

[16] Briyne ND, Atkinson J, Pokludová L, Borriello SP, Price S (2013). Factors influencing antibiotic prescribing habits and use of sensitivity testing amongst veterinarians in Europe. Vet Rec.

[17] Magalhães-Sant’Ana M, More SJ, Morton DB, Hanlon A (2016). Ethical challenges facing veterinary professionals in Ireland: results from policy Delphi with vignette methodology. Vet Rec.

[18] Magalhães-Sant’Ana M, More SJ, Morton DB, Hanlon AJ (2017). Challenges facing the veterinary profession in Ireland: 1. Clinical veterinary services. Ir Vet J.

[19] Magalhães-Sant’Ana M, More SJ, Morton DB, Hanlon AJ (2017). Challenges facing the veterinary profession in Ireland: 3. Emergency and casualty slaughter. Ir Vet J.

[20] Magalhães-Sant’Ana M, Hanlon AJ (2016). Straight from the Horse’s mouth: using vignettes to support student learning in veterinary ethics. J Vet Med Educ.

[21] Forman J, Damschroder L (2008). Qualitative content analysis. Empirical methods for bioethics: a primer (advances in bioethics; vol. 11).

[22] Wensley SP (2016). Should vets be concerned about biodiversity loss?. Vet Rec.

[23] Dean WR, McIntosh WA, Scott HM, Barling KS (2011). The role of trust and moral obligation in beef cattle feed-lot veterinarians’ contingent adoption of antibiotic metaphylaxis recommendations. Int J Sociol Agric Food.

[24] Redding LE, Cubas-Delgado F, Sammel MD, Smith G, Galligan DT, Levy MZ (2014). Comparison of two methods for collecting antibiotic use data on small dairy farms. Prev Vet Med.

[25] Hardin G (1968). The tragedy of the commons. Science.

[26] Magalhães-Sant’Ana M (2017). Resisting the urge to prescribe vancomycin. In Pract.

[27] Liu Y-Y, Wang Y, Walsh TR, Yi L-X, Zhang R, Spencer J (2016). Emergence of plasmid-mediated colistin resistance mechanism MCR-1 in animals and human beings in China: a microbiological and molecular biological study. Lancet Infect Dis.

[28] Bos MEH, Mevius DJ, Wagenaar JA, van Geijlswijk IM, Mouton JW, Heederik DJJ (2015). Antimicrobial prescription patterns of veterinarians: introduction of a benchmarking approach. J Antimicrob Chemother.

[29] EMA Committee for Medicinal Products for Veterinary Use (CVMP) and EFSA Panel on Biological Hazards (BIOHAZ). EMA and EFSA Joint Scientific Opinion on measures to reduce the need to use antimicrobial agents in animal husbandry in the European Union, and the resulting impacts on food safety (RONAFA). EFSA J. 2017;15(1). http://doi.wiley.com/10.2903/j.efsa.2017.4666. Accessed 2 June 2017. 10.2903/j.efsa.2017.4666 PMC7010070 32625259

[30] Anonymous (2016). MEPs vote to ban prophylactic use of antibiotics in animals. Vet Rec.

[31] Gibbons JF, Boland F, Egan J, Fanning S, Markey BK, Leonard FC (2016). Antimicrobial resistance of Faecal Escherichia Coli isolates from pig farms with different durations of in-feed antimicrobial use. Zoonoses Public Health.

[32] More SJ, Clegg TA, O’Grady L (2012). Insights into udder health and intramammary antibiotic usage on Irish dairy farms during 2003-2010. Ir Vet J.

[33] More SJ, Clegg TA, McCoy F (2017). The use of national-level data to describe trends in intramammary antimicrobial usage on Irish dairy farms during 2003-15. J Dairy Sci.

[34] HPRA. Report on consumption of veterinary antibiotics in Ireland during 2014. Health Products Regulatory Authority; 2015. http://www.hpra.ie/homepage/about-us/publications-forms/newsletters/item?id=e8010426-9782-6eee-9b55-ff00008c97d0&t=/docs/default-source/publications-forms/newsletters/report-on-consumption-of-veterinary-antibiotics-in-ireland-during-2014. Accessed 5 Jan 2017.

[35] Veterinary Ireland Policy Document on Antimicrobial Resistance (AMR) 2014. Veterinary Ireland National Council; 2014. http://www.veterinaryireland.ie/images/Veterinary_Ireland_Policy_on_Anti-Microbial_Resistance_2014.pdf. Accessed 16 Jan 2017.

[36] Magalhães-Sant’Ana M, More SJ, Morton DB, Osborne M, Hanlon A (2015). What do European veterinary codes of conduct actually say and mean? A case study approach. Vet Rec.

[37] VCI. Ethical Veterinary Practice. Veterinary Council of Ireland; 2008. http://www.vci.ie/Publications/Guidelines/Ethical-Veterinary-Practice. Accessed 5 Jan 2017.

[38] Craven N (2011). Antimicrobials in the balance: prudence and the precautionary principle. Vet Rec.

[39] Speksnijder DC, Jaarsma ADC, van der Gugten AC, Verheij TJM, Wagenaar JA (2015). Determinants associated with veterinary antimicrobial prescribing in farm animals in the Netherlands: a qualitative study. Zoonoses Public Health.

[40] Gibbons JF, Boland F, Buckley JF, Butler F, Egan J, Fanning S (2013). Influences on antimicrobial prescribing behaviour of veterinary practitioners in cattle practice in Ireland. Vet Rec.

[41] Postma M, Speksnijder DC, Jaarsma ADC, Verheij TJM, Wagenaar JA, Dewulf J (2016). Opinions of veterinarians on antimicrobial use in farm animals in Flanders and the Netherlands. Vet Rec.

[42] Cuny C, Friedrich AW, Witte W (2012). Absence of livestock-associated Methicillin-resistant Staphylococcus Aureus Clonal complex CC398 as a nasal colonizer of pigs raised in an alternative system. Appl Environ Microbiol.

[43] Kruse H, Johansen BK, Rørvik LM, Schaller G (1999). The use of Avoparcin as a growth promoter and the occurrence of Vancomycin-resistant Enterococcus species in Norwegian poultry and swine production. Microb Drug Resist.

[44] Bogaard VD, EA LN, Driessen C, Stobberingh EE (2001). Antibiotic resistance of faecal Escherichia Coli in poultry, poultry farmers and poultry slaughterers. J Antimicrob Chemother.

[45] Robinson TP, Wertheim HFL, Kakkar M, Kariuki S, Bu D, Price LB (2016). Animal production and antimicrobial resistance in the clinic. Lancet.

[46] Prescott JF (2014). The resistance tsunami, antimicrobial stewardship, and the golden age of microbiology. Vet Microbiol.

[47] Speksnijder DC, Jaarsma DAC, Verheij TJM, Wagenaar JA (2015). Attitudes and perceptions of Dutch veterinarians on their role in the reduction of antimicrobial use in farm animals. Prev Vet Med.

[48] van Dijk L, Hayton A, Main DCJ, Booth A, King A, Barrett DC, et al. Participatory policy making by dairy producers to reduce anti-microbial use on farms. Zoonoses Public Health. 2016; 10.1111/zph.12329. 10.1111/zph.12329 28026910

[49] A European One Health Action Plan against Antimicrobial Resistance (AMR). European Commission; 2017. http://europa.eu/rapid/press-release_IP-17-1762_en.htm. Accessed 17 July 2017.

[50] EC workshop with EMA: Data collection on consumption of veterinary antimicrobials in Europe – achievements, challenges and way forward. In: Health and Food Safety. Brussels: Directorate-General for Health and Food Safety - European Commission; https://ec.europa.eu/health/amr/events/ev_20170426_en. Accessed 17 July 2017.

[51] More SJ, Hanlon A, Marchewka J, Boyle L (2017). Private animal health and welfare standards in quality assurance programmes: a review and proposed framework for critical evaluation. Vet Rec.

[52] EIP-AGRI. EIP-AGRI Focus Group on Reducing antibiotics in pig farming: Final report. European Innovation Partnership for Agricultural Productivity and Sustainability, European Commission; 2015. https://ec.europa.eu/eip/agriculture/en/content/eip-agri-focus-group-reducing-antibiotic-use-pig-farming-final-report. Accessed 16 Jan 2017.

[53] Briyne ND (2016). Veterinary attitudes towards antimicrobial resistance. Vet Rec.

