# Supplementary material for: Challenges facing the veterinary profession in Ireland: 2. On-farm use of veterinary antimicrobials
Source: Ir Vet J. 2017 Sep 15;70:28. doi: 10.1186/s13620-017-0106-9 (PMC5602862; doi:10.1186/s13620-017-0106-9)
Supplement: Additional file 1: — Interview Guide - FOCUS GROUPS VAM. (PDF 11 kb) [file 13620_2017_106_MOESM1_ESM.pdf]

210 min (3.5 hours) | **Interview Guide - FOCUS GROUPS VAM** | TECHNICAL

Interview Guide - FOCUS GROUPS VAM

210 min (3.5 hours) — **TECHNICAL**

10.30 am — **MORNING SESSION**

1	Introductions<br>Explain the purposes of the focus group<br>Privacy, Anonymity, informed consent<br>**(10 min - 10.40)**	Thank participants for coming.<br>Present Information Sheet (in case participants haven't read it and ask for queries)<br>Ask for signed consent forms (bring additional copies)<br>The recorded tapes will be used for analysis only. Transcripts Personal data will not be used.<br>Demographic data will not be reported in such a way that your identity can be revealed.
2	**Introductions (20 min - 11.00)**	Let participants introduce themselves to create familiarity.
3	**Icebreaker**<br>**(30 min - 11.30)**	'Ice-breaker' - list 3 challenges associated with the use of antibiotics in vet/human* medicine? Participants will be asked to write their list onto post-its, without discussing with other participants.<br>The facilitator will then go around the table to ask each participant to list their issues.
4	**Practical ethical dilemma**<br>**Framing the Issues (Facts)**<br>**(60 min. - 12.30)**	Vignette: Joan routinely prescribes broad spectrum antibiotics (injectable and tubes) to a dairy farmer with a large herd of 300 animals in Co. Cork. The herd has a low record of somatic cell count (< 100 000 cells/mL). Every dry cow gets a tube and most cows are injected. "The preventive use of antibiotics has made this farm one of the best in Ireland – at the end that's good for the animals, and cheaper for the farmer."<br>How does this case relates with challenges hitherto identified?<br>Who should take responsibility for the prescription and administration of antimicrobials?

Place of interview:
Date:
Time:
Duration:

13.30 pm — **AFTERNOON SESSION**

5	**Exploring barriers to solutions**<br>**(35 min - 14.05)**	Individually: Write on card the answer to the question: What are the barriers to preventing responsible prescription and use of veterinary antimicrobials?<br>Group discussion: *Why is that? What do you mean by that? Do you all agree with this view?*
6	**Exploring Possible Solutions**<br>**(45 min - 14.50)**	Collective exercise: In your experience, what strategies could be adopted to improve the responsible prescription and use of antimicrobials?<br>**Group Discussion:** who should take the responsibility in dealing with responsible prescription of AB?<br>who should take the responsibility in dealing with the responsible administration of AB?
7	**Debriefing**<br>**(10 min - 15.00)**	a. Finish with questions: *Are there any issues related to the ethics of … that we have not dealt with* ?<br>*b.* Is there anything else that you would like to talk about concerning this meeting?<br>c. Thank participants.
